# Cross-Cultural Translation and Application of the Lieberman–Brian Inclusion Rating Scale for PE in German-Speaking Countries

**DOI:** 10.3390/ijerph19137891

**Published:** 2022-06-27

**Authors:** Martin Giese, Michelle Grenier, Lauren J. Lieberman, Stefan Meier

**Affiliations:** 1 Department of Natural and Human Sciences, Heidelberg University of Education, 69120 Heidelberg, Germany; martin.giese@ph-heidelberg.de; 2 Department of Kinesiology, University of New Hampshire, Durham, NH 03824, USA; michelle.grenier@unh.edu; 3 Department of Kinesiology, Sport Studies and Physical Education, College at Brockport, The State University of New York, Brockport, NY 14420, USA; llieberman@brockport.edu; 4 Centre for Sport Science and University Sports, University of Vienna, 1150 Vienna, Austria; 5 Centre for Teacher Education, University of Vienna, 1090 Vienna, Austria

**Keywords:** inclusion, children with disabilities, cross-cultural translation, professional preparation, teacher assessment, special education

## Abstract

Overcoming participation barriers of students with disabilities in physical education is of great importance and an internationally recognized goal. Research highlights that students with disabilities have mixed feelings about their inclusion experiences in physical education. Physical education teachers often do not feel prepared to appropriately support all students. In German-speaking countries in particular, there is a strong tradition of segregation, with varying interpretations of inclusion. In this light, an instrument to reliably assess the inclusive potential of physical education is needed, thereby providing data on the efficacy of teachers’ practices. Such an assessment scale would be important to identify barriers to inclusive physical education while providing teachers with data that could potentially enhance the learning environment. The purpose of this study was to outline initial insights into the cross-cultural translation process of the Lieberman/Brian Inclusion Rating Scale for PE in German-speaking countries. The translation process followed suggestions for transcultural validation. Expert review was provided to check content and face validity. Major item challenges centered around paraeducators, gym management, and conceptual differences regarding physical education.

## 1. Introduction

With the adoption of the UN Convention on the Rights of Persons with Disabilities (CRPD) [1] and in line with the UN guidelines for inclusion [2], the international community has committed itself to removing barriers in educational contexts. The overall goal is to provide inclusive educational opportunities for children and youth with and without disabilities. Within the context of the school, children cannot be excluded from the general education setting on the basis of disability. The normative orientation in this educational policy paradigm has been largely unquestioned in the international inclusion discourse for some time [3].

The process of moving from segregated to more inclusive environments also relates to physical education (PE). However, studies repeatedly show that students with disabilities perceive inclusive physical education (IPE) as a subject with particularly diverse barriers [4]. With respect to inclusion efforts, overcoming participation barriers of students with disabilities in physical education is of great importance. A primary concern has been students’ lack of participation and feelings of belonging [5,6]. Additionally, many students with disabilities generally have lower motor activity levels and delays in the development of their motor skills, particularly children who are visually impaired or blind [7]. In addition, physical education teachers often feel inadequately qualified to appropriately support students with disabilities in their classes [8]. Both for the development of an active lifestyle, in the sense of sustainable health education, and for participation in sports culture, in the sense of social participation, these findings are to be understood as a fundamental barrier, which should be addressed against the background of the global inclusion agenda.

However, this is countered by the fact that there is a lack of understanding within the extant literature regarding good practices as they pertain to the inclusion of students with disabilities in physical education. Teacher actions and practices are critical for fostering positive relationships, adaptations, and a safe learning environment [9]. Teachers who create positive conditions within a heterogeneous classroom are knowledgeable of students’ unique needs and their learning goals and provide both the resources and support that contribute to students’ engagement in the academic content [10,11].

In German-speaking countries, an instrument to reliably assess the inclusiveness of physical education is of critical interest, thereby providing data on the efficacy of teachers’ practices [12,13]. Such an assessment scale would be important to identify barriers to inclusive physical education while providing teachers with data that could potentially enhance the learning environment. The Lieberman/Brian Inclusion Rating Scale for Physical Education (LIRSPE) attempts to assess the effort that a PE teacher makes in developing an inclusive environment in the US [14]. However, applying a scale to the physical education setting in another culture is complex in terms of content and methodology, given the variability of teaching conditions and circumstances within each of the countries. In this next section, we present the specifics of the German school system, particularly as it relates to the education of students with disabilities. We follow that within a discussion of physical education in Germany situated in relation to the European Union (EU) context. Our efforts are aimed at expanding on the implications in the development of a scale that adequately takes into account the particular cultural characteristics in Germany in order to advance the practice of inclusion for students with disabilities.

### 1.1. Education and Inclusion of Students with Disabilities in Germany

Based on the global agenda of an inclusive society, there is an international movement to encourage equality for students with disabilities and promote inclusive, rather than segregated, education. However, strategies to promote inclusive education vary widely depending on the country in which specific conditions exist. Within the EU, each country has established its own legislation implementing inclusive education in different ways, reflecting its social, cultural, and historical contexts [15]. Given that, it is imperative to reflect on the possibilities and challenges that can occur by utilizing an evaluation scale across cultural arenas by outlining the special features of the German-language education system in the context of physical education. We acknowledge the research gap between international and German-speaking countries in physical education [16]. In particular, in pedagogical fields, German-language and international research activities in physical education exist in parallel, without systematic exchange [17].

Within the German educational system, there is a strong tradition of segregation, with varying interpretations of inclusion and corresponding school policies subject to controversial and often highly emotional discussions [18]. While Ahrbeck and Fickler-Stang [19] are advocating for the partial maintenance of special schools only for students with disabilities, Reich [20] demands the abolition of all school segregation. Although Germany is taking a special path with its segregated special needs school system, there is opposition in Germany as to how the CRPD should be interpreted. In addition to the debate on inclusion, there exist significant differences in the way students with disabilities are supported in Germany, particularly with regard to the support services that are offered within other countries. Within the US educational system, for example, adapted physical education (APE) teachers, or teachers specifically trained to work with students with disabilities, support Physical Education teachers in making curricular adjustments and offering equipment choices. In many cases, students with disabilities have paraeducators assigned to them as support personnel within the PE class. Paraeducators work with Physical Education teachers to support students’ engagement in gymnasium activities. In Germany, neither APE teachers nor paraeducators exist as a support service in Physical Education. The vision of an inclusive society within an inclusive educational system is a long-term goal for both the school system and society at large in Germany.

Additionally, the German educational system utilizes a categorical approach in distinguishing between different types of impairment. The official types of impairment in the German educational system are (in order of occurrence): learning disorders, intellectual disabilities, behavioral disabilities, language impairments, physical disabilities, hearing impairments, and visual impairments. Each of the eight types of impairment has its own school. For example, a student with a visual impairment can attend a special school for people with visual impairment or blindness. If students with disabilities attend general education schools, they receive support by visiting teachers from the students’ support services that are typically located in the special schools. On the basis of an expert report from a special education teacher, a determination is made on a case-by-case basis as to the level of support provided for each student with a disability. School laws within each of the federal states determine how much support the student is entitled to. Typically, the student is assigned a special education teacher for a limited number of hours per week, tasked with supporting the student in class, as well as providing consultation to the general education teacher on how best to support the student’s academic success. However, it is critical to note that support services are generally focused on the major subjects (German, Mathematics, and English), while support in Physical Education is not usually provided [21].

In Germany, 44.48% of all learners needing special education in 2020 received inclusive education in their local school in primary and lower-secondary education. In the category of vision impairment (VI), for example, it was as high as 51.0%. However, it should be noted that this value varies greatly depending on the federal state and age of the child. In the federal state Schleswig-Holstein, for example, 100% of all students with VI attend general schools, while in the state of Hessen, this figure is only 15.5% [22].

At present, almost all federal states, which are solely responsible for education policies in Germany, continue to prefer—particularly in the case of sensory disabilities—to maintain two educational systems [23], despite the UN Committee on the Rights of Persons with Disabilities [24] explicitly declaring that the full realization of article 24 (CRPD) “is not compatible with sustaining two systems of education: mainstream and special/segregated education systems” [1] (para 39). Thus, parents of students with disabilities continue to have the choice as to whether whether their child will attend an inclusive school or a special school, which is deemed a parental voting right. Germany and Austria are EU countries with a strong tradition of segregating children with disabilities and relatively high cultural resistance against inclusive efforts. In accordance with this, inclusion rates are relatively low in Germany, garnering continued criticism of the education system (fundamental criticism is formulated, for example, in the UN Special Report on the Right to Education [25] and within the State Party Review by the UN Committee on the Rights of Persons with Disabilities [26].

### 1.2. The German Concept of Physical Education within the EU Context

Social, cultural, and historical contexts account for a variety of physical education terminology and concepts within the EU. Naul [27] structured and termed four main vectors of physical education based on the varying cultures and traditions. For example, Germany traditionally focuses on “sport education”, while the Netherlands has a focus on “movement education” [27] (p. 47). With respect to Germany, Physical Education is fundamentally based on a dual mission (known as *Doppelauftrag*). It promotes the notion of learning in and through physical education, targeting students’ personal development as well as the development of sport-specific competence [28]. This concept originated in Germany in 2000 and has been adopted by other EU countries, including Austria [29] Luxembourg [30], and Switzerland [31].

Within the sport education approach, individual experience and personal meaning are encouraged through the instructional principle of the “multiperspectivity” of sporting experiences. This multi-perspective education enables students to acquire new learning experiences apart from established sporting pathways. These may include discovering physical activity and sports in the context of risk, creativity, self-expression, enjoyment, impression and sensation, health, or performance. Such didactic approaches align with concerns of diversity [32]. Similarly, the Society for Health and Physical Education [33] identified five standards for what students should know and be able to do as a result of their participation in physical education. In particular, standards 4 and 5 identify personal responsibility, positive social interactions, and expression as primary goals. Thus, within Physical Education settings, the curricular content and instructional practices of Physical Education teachers are designed to foster the development of competencies associated with enjoyment, expression, and creativity.

### 1.3. Assessing the “Inclusiveness” of Physical Education—State of Research

As we have described, the discourse on inclusive education is unclear across international contexts [34]. We will argue that at the forefront of these practices are teachers’ direct actions that can foster equitable practices aimed at improving educational experience for all children. This is in line with Booth and Ainscows’s “Index for Inclusion” [35], which highlights teachers as key actors in creating inclusive cultures, establishing inclusive structures, and developing inclusive practices. Accommodating the needs of students with disabilities through meaningful engagement is essential in creating an inclusive environment [36]. Given the cultural differences that exist on an international scale to advance an inclusive agenda, research is needed that explores effective inclusive practices within the Physical Education setting [37]. Within the context of German schools, an assessment tool is still missing to provide Physical Education teachers with feedback evaluating the extent to which the PE teacher make an effort to ensure that the classes are inclusive for all children.

## 2. Methods

The English-designed Lieberman/Brian Inclusion Rating Scale was the primary instrument used in this study [14]. Within the domain of international research, translation is important, especially if the items are to be consistently understood by raters and participants. This is of particular importance in the context of cross-cultural backgrounds and educational contexts. In order to translate and to adapt the LIRSPE (both scale and rubrics) to the cultural conditions that exist in Germany, a two-step, cross-translation procedure was employed.

### 2.1. Step I

Our first steps included the translation of the LIRSPE from its original language—English—to German, following the suggestions made by Banville et al. [38] for trans-cultural validation (forward translation by two native German-speaking APE professionals who are also familiar with English). In this process, items were discussed with the authors of the LIRSPE, and adaptations were made, according to contextual referencing. Next, the German version received a backward translation by professional English language services. The goal of the translation procedure was to secure accurate meaning of the terminology.

### 2.2. Step II

An expert review was provided to check content and face validity, clarity, and conciseness, as well as relevance for teaching Physical Education to make sure the test represented all facets of what it is purported to measure [39]. In order to address this, a panel of experts participated online via Delphi techniques [40,41]. German-speaking experts were asked to provide a judgment on the coherence of the translation (5-point Likert scale), a judgment on the extent to which the item was professionally relevant, to rate the potential for inclusive Physical Education (5-point Likert scale, only for the LIRSPE), and to provide general feedback and suggestions on every single item on the scale. After applying these two steps to the LIRSPE, the same procedure was administered to the LIRSPE rubrics (excluding judgment of relevance).

Experts (*n* = 8, all professors in PETE programs and with more than 9 years of experience in the field) rating the LIRSPE were different from those judging the LIRSPE rubrics to double check content and face validity. All experts were German-speaking professors in PETE programs and faculty who had published research regarding inclusion, possessed a doctorate degree in Physical Education, and possessed experience in the field. Experts rating the LIRSPE (*n* = 5) were between the ages of 35 and 66 years (M = 49.3) and possessed 9.2 years of experience on average. Experts judging the LIRSPE rubrics (*n* = 3) were between the ages of 43 and 66 years (M = 57.7) with approximately 11.3 years of experience.

## 3. Results

### 3.1. Scale

**Figure 1:**
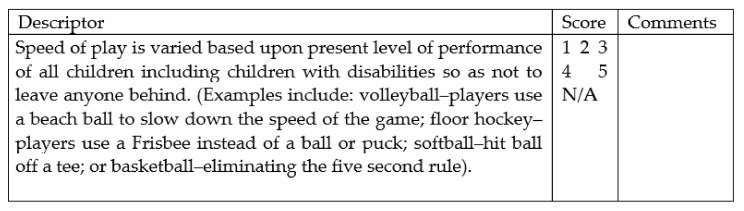
Original LIRSPE item 4 [14].

We report the ratings on the LIRSPE and rubrics step by step, as it was organized within the study. First, the coherence of the translation of the scale was highly rated by the experts (M = 4.46, SD = 0.39). Three items did not score above four out of five (original LIRSPE items 2, 4, and 22). Major critiques included issues of clarity in terminology. For example, experts pointed to “beach ball” (speed of play within the lesson, see Figure 1), which is not well-suited to the German context: “I recommend to delete this” (expert 4).

Thus, we discussed whether we needed the same number of examples for each item and decided to choose the ones that best exemplified the item. As there were no comments to better illustrate items—contrary to the original study—we only used the example “basketball–eliminating the five second rule”, which is commonly known in the German context. In addition, experts commented on the item “Noise and distractions are reduced to maximize success”, which led to further discussion with the authors from the original LIRSPE to clarify the meaning. With feedback provided by the experts (e.g., expert 2: “is ‘maximize success’ related to learning outcomes?”), we were able to improve the German wording.

**Figure 2:**
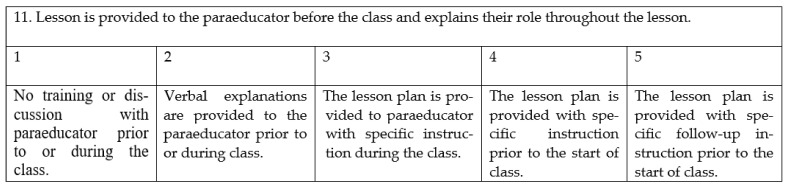
Original LIRSPE item and rubric 11 [14].

*Regarding the extent to which the items of the LIRSPE were eligible to rate the potential for inclusion in physical education, expert ratings ranged between 3.00 and 5.00 (M = 4.25, SD = 0.54), and six items did not score above four out of five (original LIRSPE items 16, 17, 18, 22, 24, and 27). Major critiques (four out of these six items) centered around a term most of the experts considered management issues. For example, within Game/Activity—Team Sport (“Teacher avoids students waiting in line”, original LIRSPE item 17), experts mentioned that this was a kind of “normal” classroom management and not specific to inclusive Physical Education: “[…] belongs to a gym-management probably, but I think it is not specific for inclusion” (expert 5). A fundamental difference is the item of the paraeducator (see Figure 2 for an example item), a role that does not exist in Germany, nor is there a similar support system: “some items presuppose a paraeducator, whereas in Germany this is often not the case” (expert 3).*

### 3.2. Rubrics

Applying these steps to the rubrics, experts rated the coherence of the translation highly (M = 4.64, SD = 0.29). The overall mean improved compared to the ratings of the LIRSPE. However, 7 rubrics (out of 140) did not score above four out of five (original LIRSPE rubrics 11.1, 13.3, 18.2, 18.3, 26.2, 26.3, and 26.4) and were commented on by all experts. Feedback and minor critique centered on the use of paraeducators and gym management. For example, the experts highlighted “No training or discussion with paraeducator prior to or during the class” (use of paraeducator, see Figure 2), as this role does not exist in German-speaking countries: “term support staff/paraeducator: check qualification with authors from original scale” (expert 2). After checking, we decided to leave in these items, but using the term “assistant teacher”. Even if not equivalent, this is known in Germany and in the direction of the support system “paraeducator”.

Another comment emphasized the generic goal of Physical Education, in particular, the focus on skill performance/motor learning.

## 4. Discussion

The purpose of this study was to investigate the extent to which a rating scale based on the LIRSPE [14] could be applied to the German-speaking context. To the best of our knowledge, no assessment tool exists to provide Physical Education teachers with feedback evaluating the extent to which Physical Education classes are inclusive for all children in German-speaking countries. This paper outlines the first steps with attention to the cross-cultural challenges in translating the LIRSPE within the German-speaking educational system, which has a particularly strong tradition of segregation.

With respect to face and content validity, the results indicated the high coherence of the translation for both the LIRSPE and rubrics. Experts rated each item very highly, with low variance among reviewers. Regarding the extent to which the items were likely/suitable to rate the potential for an inclusive Physical Education environment, experts rated each item highly (greater than four). However, some items were not judged above a four (out of five). In addition, experts provided feedback and comments indicating minor (clarity of wording) and major challenges (context) for both the scale and the rubric. With regard to clarity of wording, there were some examples that did not fit well to the German context. Hence, we reduced examples to illustrate items. In contrast, concrete scenarios and examples were suggested by the experts within the validation study of the original LIRSPE version. Such context challenges are more at the surface, whereas others go beyond.

The major challenges centered around (1) paraeducators, (2) gym management, and (3) conceptual differences regarding physical education. Since there are neither paraeducators nor a similar support system in Germany (1), it is not surprising that the raters identified major challenges regarding this issue. Currently, the translation follows the idea of presenting a German term that may provide an appropriate idea of paraeducators as understood in the US. However, given the unfamiliarity of the term as well as the practice of using paraeducators, there exists no comparable term. Further research must show whether the translation will lead to valid results. As it is evident that paraeducators improve the inclusiveness of Physical Education, our goal is to leave the items related to paraeducators in the rating scale. Within the meaning of Article 8 of the CRPD (awareness raising), we want to raise awareness of the need for paraeducators or a support system to implement. Experts also expressed concerns on aspects including classroom management, which they highlighted as gym management (2). They mentioned that these aspects were “normal/regular” for PE lessons and not specific to inclusive Physical Education. However, future research should elaborate on aspects specific to the German context by adding items reflective of German principles, such as multi-perspectivity [32].

Additionally, experts commented heavily on items focusing on skill performance and motor learning. Their critique centered around the main goal of Physical Education (3). From a conceptual point of view (see in Introduction), Physical Education in Germany promotes learning through the development of sport-specific competences and students’ personal development [28]. Thus, the heavy focus on the value of skills and performance was considered a limitation to an inclusive environment. Our translation aligns with the original LIRSPE as highly skill-focused. On the one hand, this serves as a good foundation for comparative studies, but on the other hand, it minimizes the German focus on personal development. As it is our intention to prioritize both, adding items reflecting the conceptual ideas of Physical Education in Germany would be useful.

### 4.1. Limitations

Despite the strengths inherent within the results (e.g., strong validity scores), our study has several limitations, in particular, the extent to which the scale reflects the German context. Future research should cross-check such contextual issues through the addition of a Delphi method. Items can then be added, focusing on conceptual ideas specific to inclusive Physical Education in the German context. It would be helpful to have Physical Education teachers involved in this process to check for “practical aspects” of the scale. As we want to conduct comparative studies with the original LIRSPE, this strategy for complementary contexts serves as a good foundation. The add-on items for the German context (or another) provide a specific addition, whereas the original LIRSPE items remain the same and enable comparative studies (i.e., the US versus Germany).

### 4.2. Implications for the Practice

The results of this study reveal insights into the cross-cultural translation and validation process of the LIRSPE in German-speaking countries, reflecting the multi-layered cultural complexity. Currently, there exists no tool in German-speaking countries to provide an assessment on the extent to which Physical Education teachers provide an inclusive environment for all children. Teacher feedback evaluating the efforts made by educators will support the global agenda of inclusion. Teachers and practitioners may choose to use the scale for reflection and self-evaluation with an outside rater or as their own intervention tool to improve inclusive practices within a PE setting.

### 4.3. Future Research

Future research should focus on reliability and explore whether an existing factor structure is present. This is important (a) to show that the scale is reliable across outside raters so that when evaluated, teachers can be assured of reliable results. Furthermore, (b) if both teachers and students show high or low marks on the same items, then the scale demonstrates construct validity. Future research should address these next steps in the validation process. In addition, future research should cross-check such contextual issues through the addition of a Delphi method. Other future research may investigate the scale’s reliability and assess teachers’ practices on the extent to which children felt included in the Physical Education class. Examining students’ ratings concurrently with teachers’ ratings of inclusion in future research projects would offer tangible indications of teachers’ efforts. Construct validity may also be examined.

## Data Availability

The data presented in this study are available on request from the corresponding author.
